# Identification of Brain Nuclei Implicated in Cocaine-Primed Reinstatement of Conditioned Place Preference: A Behaviour Dissociable from Sensitization

**DOI:** 10.1371/journal.pone.0015889

**Published:** 2010-12-29

**Authors:** Robyn Mary Brown, Jennifer Lynn Short, Andrew John Lawrence

**Affiliations:** 1 Florey Neuroscience Institutes, University of Melbourne, Parkville, Victoria, Australia; 2 Monash Institute of Pharmaceutical Sciences, Parkville, Victoria, Australia; 3 Centre for Neuroscience, University of Melbourne, Parkville, Victoria, Australia; Centre for Neurogenomics and Cognitive Research, The Netherlands

## Abstract

Relapse prevention represents the primary therapeutic challenge in the treatment of drug addiction. As with humans, drug-seeking behaviour can be precipitated in laboratory animals by exposure to a small dose of the drug (prime). The aim of this study was to identify brain nuclei implicated in the cocaine-primed reinstatement of a conditioned place preference (CPP). Thus, a group of mice were conditioned to cocaine, had this place preference extinguished and were then tested for primed reinstatement of the original place preference. There was no correlation between the extent of drug-seeking upon reinstatement and the extent of behavioural sensitization, the extent of original CPP or the extinction profile of mice, suggesting a dissociation of these components of addictive behaviour with a drug-primed reinstatement. Expression of the protein product of the neuronal activity marker *c-fos* was assessed in a number of brain regions of mice that exhibited reinstatement (R mice) versus those which did not (NR mice). Reinstatement generally conferred greater Fos expression in cortical and limbic structures previously implicated in drug-seeking behaviour, though a number of regions not typically associated with drug-seeking were also activated. In addition, positive correlations were found between neural activation of a number of brain regions and reinstatement behaviour. The most significant result was the activation of the lateral habenula and its positive correlation with reinstatement behaviour. The findings of this study question the relationship between primed reinstatement of a previously extinguished place preference for cocaine and behavioural sensitization. They also implicate activation patterns of discrete brain nuclei as differentiators between reinstating and non-reinstating mice.

## Introduction

A central problem facing the treatment of drug addiction is the enduring vulnerability to relapse displayed by users despite months or even years of abstinence [1], [2]. Even with successful detoxification and the most sincere of intentions during abstinence, relapse can be an insurmountable challenge for many addicted individuals [3]. Indeed, up to 90% of addicted individuals relapse to drug-taking within 12 months of abstinence [4]. Drug craving commonly precipitates relapse and has been described as the subjective affective state experienced by humans which motivates them to seek out drugs [5], [6]. Craving for drugs can be induced in addicted individuals by exposure to drug-related paraphernalia, images or environmental contexts [7], [8]. It is the repeated exposure to such cues and contexts during the initiation and maintenance of drug use which is thought to result in these cues acquiring incentive motivational and conditioned reinforcing value [9]. Once formed, these pathological associations may ultimately contribute to the precipitation of craving and relapse upon re-exposure to drug-associated stimuli.

A variety of increasingly sophisticated animal models have provided invaluable means for understanding the neurobiology of addiction and the actions of drugs of abuse. One example is the reinstatement model of drug-seeking which is proposed to be a model of craving and relapse [6], [10], [11], [12]. This model exhibits elements of construct validity in the sense that factors which precipitate craving and relapse in humans such as cues and stress also cause relapse-like behaviour in rodents [9], [10], [11], [13]. The reinstatement model has also shown predictive validity as drugs which are currently prescribed for relapse prevention decrease drug-seeking in rodents [14], [15], [16]. The vast majority of existing studies utilising this model have examined the reinstatement of a previously extinguished operant response in order to assess drug-seeking (i.e. relapse-like) behaviour [11].

Conditioned place preference (CPP) is a model commonly used to study the rewarding and incentive motivational effects of drugs and drug-paired stimuli [17]. About a decade ago it was demonstrated that, similar to an operant response, preference for a drug-paired environment can be extinguished and subsequently reinstated by drug priming injections [18], [19]. Drug-primed reinstatement of CPP is thought to reflect renewed incentive value of the environmental stimuli via the incentive motivational effects of the prime [18]. Drug priming injections have been shown to reinstate CPP in animals previously conditioned with cocaine [18], morphine [19], amphetamine [20], nicotine [21] and ethanol [22]. Reinstatement of CPP can also be elicited by stressors such as intermittent footshock [23], conditioned fear stimuli [24] and immobilisation stress [25].

The neurobiology underlying the reinstatement of drug-seeking in an operant paradigm has been thoroughly investigated [11], [26]. In contrast, relatively little is known about the anatomical substrates involved in reinstatement of CPP and quite often the two paradigms are deemed isomorphic, with results considered directly comparable [27]. This is despite the fact that as yet no systematic neuroanatomical evaluation of this paradigm has been performed. The current study aims to rectify this situation by analysing the expression of the neuronal activity marker Fos to investigate the neuroanatomical substrates underlying primed reinstatement of cocaine-induced CPP.

Fos is the protein product of the immediate-early gene *c-fos* [28], [29] and is thought to be a marker for stimulus-elicited brain activity [30], [31]. The putative neural circuitry involved in the incentive motivational effects of cocaine-associated stimuli has been investigated previously using Fos. Thus, Fos protein expression is transiently increased by cocaine administration [32], [33], [34], exposure to cocaine-associated environmental cues [35], [36], [37] or discriminative stimuli that signal cocaine availability [38]. More recently a study was published which examined Fos protein expression resulting from cue-elicited reinstatement of extinguished cocaine-seeking behaviour [39].

As yet, no studies exist which have examined Fos expression resulting from a cocaine-primed reinstatement of CPP. This is probably due to the potentially confounded nature of this paradigm, as drug is administered prior to the reinstatement session and due to the repeated nature of drug administration during the conditioning period animals may also exhibit sensitization during this test session. By examining the reinstatement propensity of individual mice in the current study, we have identified a subgroup that did not exhibit reinstatement in response to a cocaine prime. This is despite displaying robust original CPP to cocaine, and a subsequent successful extinction of this CPP. The situation therefore provides two cohorts of mice that have been through the same experimental procedure and allows for an ideally-controlled comparison of Fos expression in the brains of these two cohorts of mice in order to elucidate, for the first time, the brain regions that are implicated in this behaviour.

## Results

### Behaviour: comparisons between reinstating and non-reinstating mice

**Figure 1:**
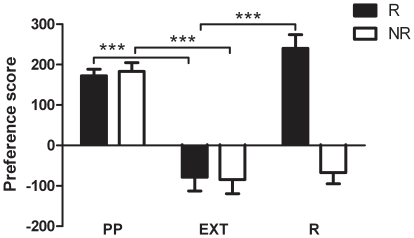
Reinstatement of conditioned place preference in reinstating (R) and non-reinstating (NR) mice (*n* = 11–16 per group). The preference score is shown over the three time points; the place preference (PP) test day, the test day following extinction (EXT) and reinstatement day (R). Preference score  =  time spent in drug-paired side minus time spent in saline-paired side on test day; *** *p*<0.001 compared to preference score after extinction for that group (two-way ANOVA with *post hoc* SNK multiple comparison test).

The two subgroups examined in this study were defined by either the absence or presence of reinstatement of CPP in response to a priming dose of cocaine (10 mg/kg, *i.p.*) on the test day following the confirmed extinction of the CPP. Reinstatement was defined a positive difference of 60 s or greater between the time spent in the cocaine-paired compartment compared to the saline-paired compartment. Figure 1 demonstrates the difference between the reinstating (R) and non-reinstating (NR) mice in terms of reinstatement behaviour. R mice displayed a robust reinstatement of place preference compared to the last day of extinction as measured by the relative increase in their preference score whereas NR mice did not. Therefore, analysis of preference score data (time spent in drug-paired compartment minus time spent in saline-paired compartment) by two-way analysis of variance (ANOVA) revealed a significant interaction between reinstatement status (i.e. R or NR) and test session (i.e. last day of extinction versus reinstatement day (F_(2,50)_  = 17.068, *p*<0.001). Student Newman-Keuls (SNK) *post hoc* analyses revealed that for R mice the preference score from the reinstatement test session was significantly higher than that on the last day of extinction (q = 11.508, *p*<0.001), demonstrating robust reinstatement, whereas for NR mice no difference was observed (q = 0.513, p>0.05). These *post ho*c analyses also reported a significant difference between R and NR mice (q = 6.029, *p*<0.001), with a difference in the preference score observed for the reinstatement test session only (q = 10.212, *p*<0.001).

Upon determining the existence of two groups of mice that differed in terms of their reinstatement behaviour it then became a priority to ascertain any other possible differences which could potentially explain their disparate reinstatement propensity. Thus, a retrospective analysis was undertaken of available data from the habituation, conditioning, extinction and reinstatement sessions. Behavioural examination of these two groups of mice is important for two reasons. Firstly, any differences between R and NR mice in terms of any other behavioural parameters could indicate a potentially significant relationship between the behaviour being measured and reinstatement behaviour. Secondly, in terms of interpreting immunohistochemical data, it is important that activation of brain regions can be attributed to the differences in reinstatement behaviour and not to any other observed behavioural difference.

**Figure 2:**
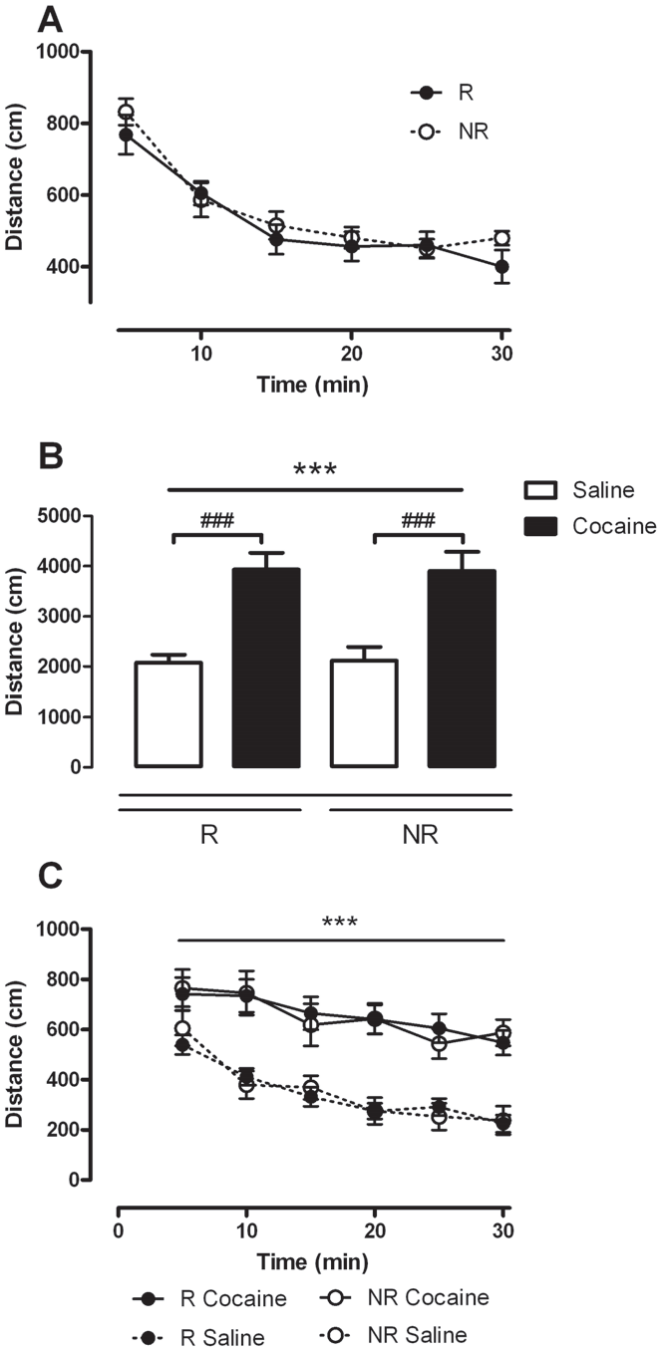
Locomotor activity in mice reinstating (R) and non-reinstating (NR) mice (*n* = 11–16 per group). (a) Locomotor activity during the 30 min habituation session. Data are expressed as the mean distance moved in cm measured in 5 min time bins over the 30 min session (± SEM). (b) Response to acute cocaine administration (20 mg/kg, *i.p.*) during the first conditioning session. Data are expressed as the mean of distance moved (cm) for the 30 min session (± SEM); *** *p*<0.001 main effect of treatment, ### *p*<0.001 compared to saline (two-way ANOVA with SNK post tests). (c) Time course data for first conditioning session. Data are expressed as the mean distance moved in cm measured in 5 min time bins (± SEM); *** *p*<0.001 main effect of treatment and time bin (three-way ANOVA).

We began by examining the locomotor data from the initial habituation session in order to determine whether any differences in response to novelty existed. As can be seen in figure 2a, habituation to a novel environment was similar between the two groups as analysed by two-way ANOVA with time bin and group as factors. Both groups displayed a similar decrease in time over the 30 min period. Hence a main effect of time bin was observed (F_(5,120)_  = 42.845, *p*<0.001) but not group (*p*>0.05).

**Figure 3:**
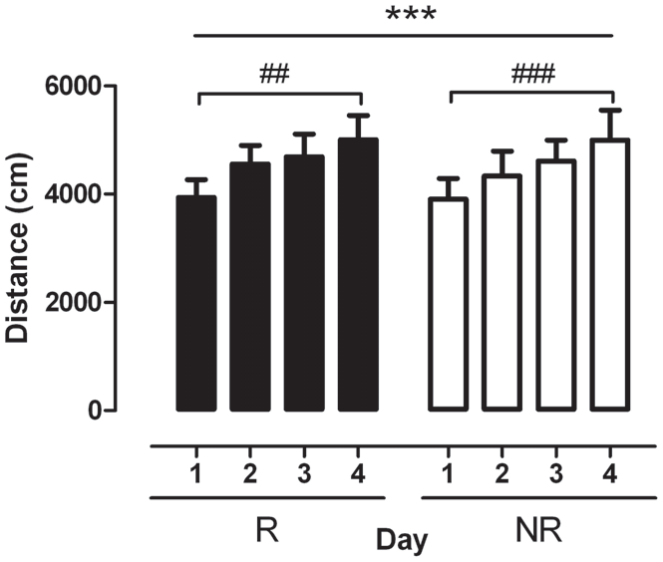
Development of sensitization during the conditioning period in reinstating (R) and non-reinstating (NR) mice (*n* = 11–16 per group). Bars represent locomotor activity in response to cocaine administration on each day of cocaine conditioning. Data are expressed as the mean of distance moved (cm) for the 30 min sessions (± SEM); *** *p*<0.001 main effect of time, ## *p*<0.01, ### *p*<0.001 compared to day 1 (two-way ANOVA with SNK post tests).

The locomotor response to acute cocaine on the first day of conditioning was then examined in order to determine if a differential existed in terms of sensitivity to the psychostimulant. As shown in figures 2b and c, there was no difference between R and NR mice in terms of their acute locomotor response to cocaine. Both groups displayed increased locomotor activity in response to acute cocaine (20 mg/kg, *i.p.*), moving more in total over the 30 min session as compared to saline (figure 2b,c). Two-way ANOVA revealed a significant effect of treatment (F_(1,25)_  = 66.963, *p*<0.001) but not group (*p*>0.05) and SNK post tests revealed the distance moved in response to cocaine was significantly greater than the response to saline in both R (q = 9.255, *p*<0.001) and NR (q = 7.360, *p*<0.001) mice (see figure 3b). This was supported by the time course data. Analysis by three-way ANOVA revealed a significant effect of time (F_(5,250)_  = 41.769, *p*<0.001) and treatment (F_(1,50)_  = 38.253, *p*<0.001) but not group (*p*>0.05).

The response to cocaine over the four conditioning days was also assessed in order to ascertain whether a similarly progressive enhancement to the locomotor activating properties of cocaine occurred in the two groups, representing development of sensitization. As can be seen in figure 3 both R and NR mice showed a similarly progressive increase in the locomotor activity generated by the same dose of cocaine over the 4 administration sessions. Analysis by two-way ANOVA revealed a significant effect of day (F_(3,75)_  = 6.470, *p*<0.001) but not group (*p*>0.05). SNK *post hoc* analysis revealed that the locomotor response to acute cocaine was significantly higher on day 4 of administration compared to day 1 for both R (q = 4.709, *p*<0.01) and NR (q = 6.088, *p*<0.001) mice.

**Figure 4:**
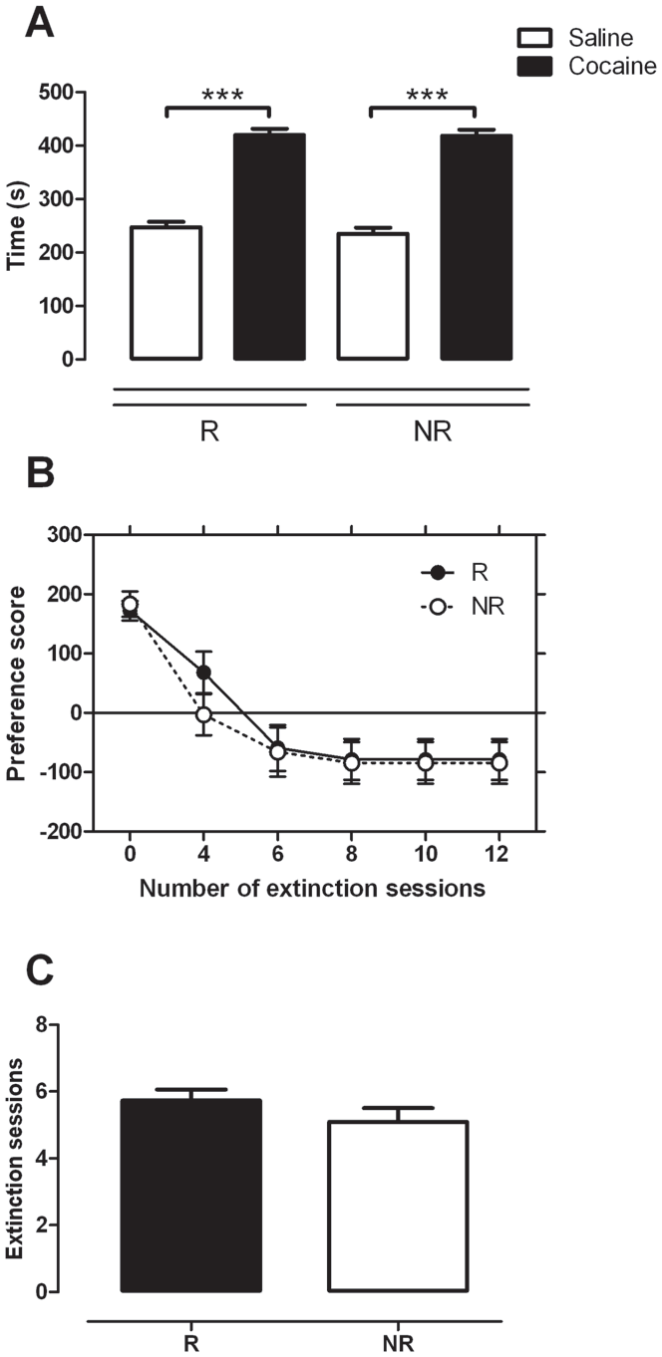
Conditioned place preference (CPP) and extinction of this CPP in reinstating (R) and non-reinstating (NR) mice (*n* = 11–16 per group). (a) CPP to cocaine (20 mg/kg, *i.p.*). The data are expressed as mean (± SEM) of the time spent in the specified cocaine- or saline-paired compartment on the test day; *** *p*<0.001 compared to saline (two-way ANOVA with SNK post tests). (b) Cumulative decrease in preference score over the course of extinction training (preference score  =  time spent in drug-paired side minus time spent in saline-paired side on test day). Data are expressed as mean (± SEM) of the cumulative preference score at each test point. (c) Average number of extinction conditioning sessions taken for mice to extinguish. Data are expressed as mean (± SEM).

With no difference found between R and NR mice in terms of their locomotor response to novelty, acute or repeated cocaine administration, the next logical step was to examine CPP data. No difference was observed between R and NR mice in terms of initial CPP to cocaine (20 mg/kg, *i.p.*) or extinction of this CPP. As can be seen in figure 4a, both R and NR mice displayed a robust preference for the cocaine-paired side during the initial PP test session. Two-way ANOVA revealed a significant effect of side (cocaine versus saline) (F_(1,25)_  = 180.109, *p*<0.001) but not group (*p*>0.05). SNK post tests revealed that the time spent in the cocaine-paired side was significantly greater than the time spent in the saline paired side for both R (q = 14.400, *p*<0.001) and NR (q = 12.715, *p*<0.001) mice. In addition, as Figure 4b demonstrates, the time course of extinction between the two groups was virtually identical, with both NR and R mice decreasing their preference score from positive to negative in a similar fashion over the extinction period. Two-way ANOVA revealed a significant effect of time (F_(5,125)_  = 35.384, *p*<0.001) but not group (*p*>0.05). Consequently there was no difference found between the two groups in terms of the average number of extinction sessions required to reach extinction criteria as analysed by Mann-Whitney Rank Sum Test (*p*>0.05, see figure 4c). Successful extinction of both R and NR mice was also revealed by *post hoc* comparisons of the reinstatement time course data (figure 1). Preference score on the last day of extinction was significantly decreased for both R and NR mice as compared to their original preference score (R: q = 9.045, *p*<0.001; NR: q = 8.011, *p*<0.001; see figure 1).

**Figure 5:**
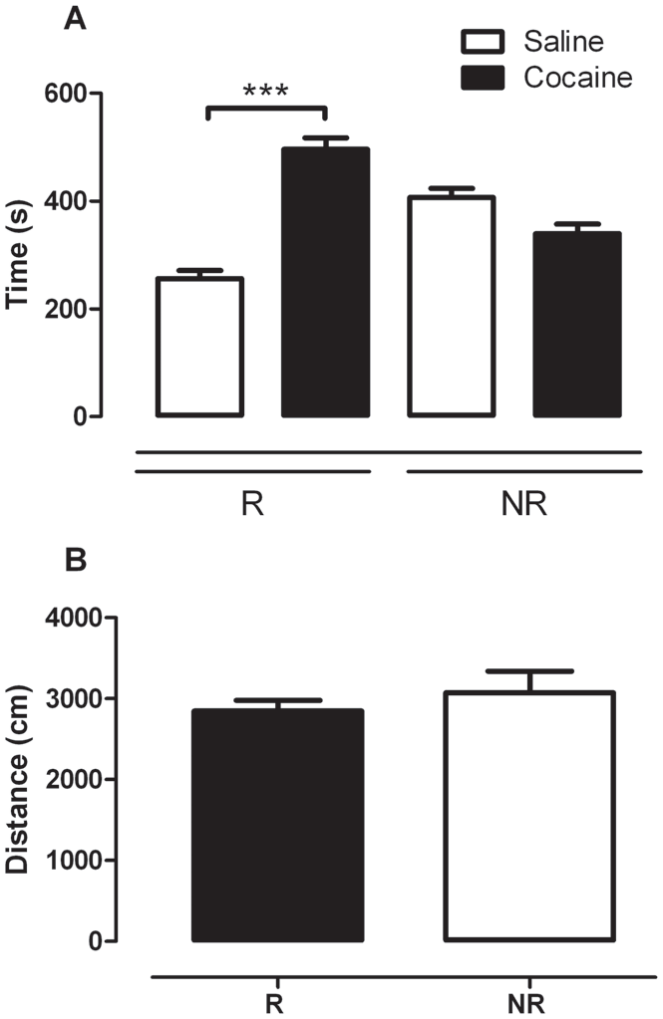
Comparison of reinstating (R) versus non-reinstating (NR) mice during the reinstatement test session following a priming dose of cocaine (10 mg/kg, *i.p.*) (*n* = 11–16 per group). (a) Preference for the cocaine-paired side compared to preference for the saline paired side during the reinstatement session. The data are expressed as mean (± SEM) of the time spent in the specified cocaine- or saline-paired compartment on the test day; *** *p*<0.001 compared to saline (two-way ANOVA with SNK post tests). (b) Locomotor activity during the reinstatement session. Data are expressed as the mean of total distance moved (cm) for the 15 min session (± SEM).

Thus, despite thorough analyses of all available behavioural parameters, no difference was found between R and NR mice apart from that found during the reinstatement session. Figure 5a illustrates the difference between R and NR mice in terms of time spent in the drug-paired side versus time spent in the saline-paired side during the reinstatement test session. Analysis by two-way ANOVA revealed a significant interaction between reinstatement status and side (time spent in either the cocaine- or saline-paired side) (F_(1,25)_  = 43.452, *p*<0.001). SNK *post hoc* analysis revealed that the time spent in the cocaine-paired side was significantly higher than that spent in the saline-paired side for R mice (q = 11.415, *p*<0.001). This was not the case for NR mice, where *post hoc* analyses revealed no significant difference between the time spent in the saline- and cocaine-paired sides (*p*>0.05). Interestingly, despite their differing CPP during the reinstatement session, there was no difference between the two groups in terms of the total distance moved as assessed by a t-test (*p*>0.05; figure 5b).

**Figure 6:**
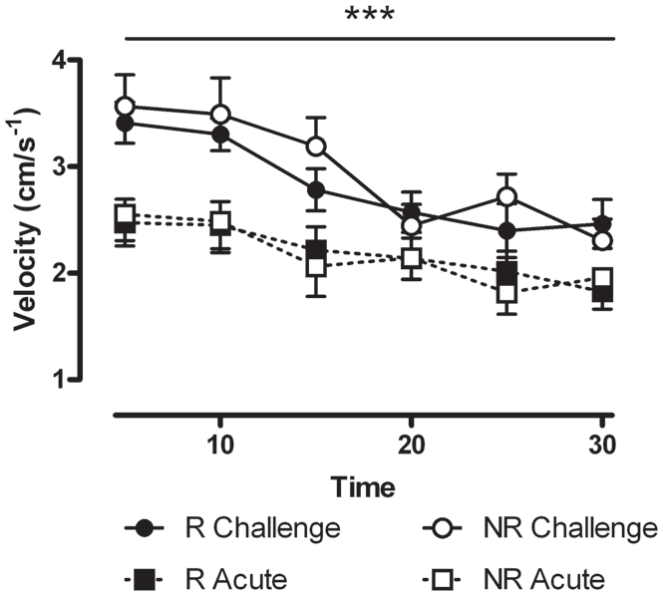
Expression of locomotor sensitization in reinstating (R) and non-reinstating (NR) mice (*n* = 11–16 per group). Locomotor activity in response to cocaine challenge (10 mg/kg, *i.p.*) versus acute cocaine (20 mg/kg, *i.p.*) on the first conditioning session. Data are expressed as the mean (± SEM) velocity (cm/s^−1^) measured in 5 min time bins over the 30 min period; ***p<0.001 main effect of treatment and time bin as factors (three-way ANOVA).

Expression of sensitization requires exposure to a challenge dose of a drug after a period of withdrawal subsequent to repeated drug administration. Repeated administration of cocaine occurs during the conditioning component of a CPP protocol. Extinction represents a period of time where no drug is administered (withdrawal). The drug prime which precipitates reinstatement is equivalent to a challenge dose of cocaine, thus the locomotor data obtained from the reinstatement session can also be used to measure expression of sensitization when compared to acute cocaine administration (day 1 of conditioning). As evident in figure 6, an enhancement of locomotor activity was observed in response to a priming dose of cocaine in both R and NR mice during the reinstatement session, demonstrating a similar expression of sensitization in both groups. Analysis of data for the 30 min session by three-way ANOVA revealed a main effect of treatment (F_(1,300)_  = 58.833, *p*<0.001) and time (F_(5,300)_  = 10.389, *p*<0.001) but not group (*p*>0.05).

**Figure 7:**
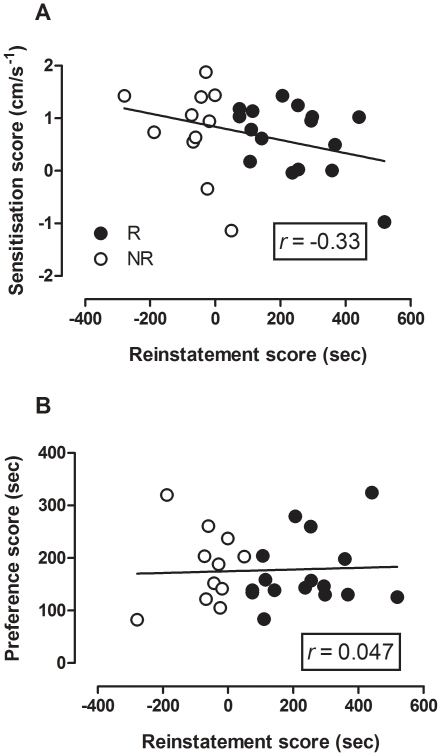
Scatter plots showing lack of correlation between reinstatement behaviour and other behaviours in R (black circles) and NR (white circles) mice (*n* = 27). (a) Reinstatement behaviour versus sensitization behaviour. Reinstatement preference score  =  time spent in drug-paired side minus time spent in saline-paired side on test day (sec). Sensitization score  =  mean velocity during challenge session minus mean velocity during day 1 of cocaine treatment (cm/s-^1^). (b) Reinstatement CPP versus original CPP. Reinstatement preference score and original preference score  =  time spent in drug-paired side minus time spent in saline-paired side on test day (sec).

In order to determine the nature of the relationship between reinstatement behaviour and either initial CPP or expression of sensitization, correlation analysis was undertaken. Pearson test revealed no significant correlation between the extent of sensitization (as measured by the change in mean velocity between acute and challenged groups) and the extent of reinstatement (*r* = −0.33, *p*>0.05) (see figure 7a). Pearson test also revealed there was no correlation between the extent of reinstatement and the extent of original CPP (*r* = 0.047, *p*>0.05) (see figure 7b).

### Immunohistochemistry: comparisons of Fos expression between reinstating and non-reinstating mice

**Figure 8:**
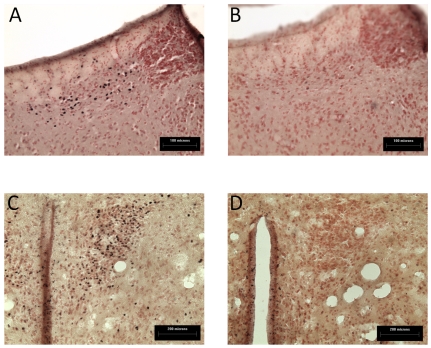
Photomicrographs of Fos-positive neurons in representative reinstating mice (A and C) and non-reinstating mice (B and D) from the lateral habenula (bregma -1.46; scale bar 100 µm) and the paraventricular nucleus of the hypothalamus (bregma -0.70-0.94; scale bar, 200 µm).

**Figure 9:**
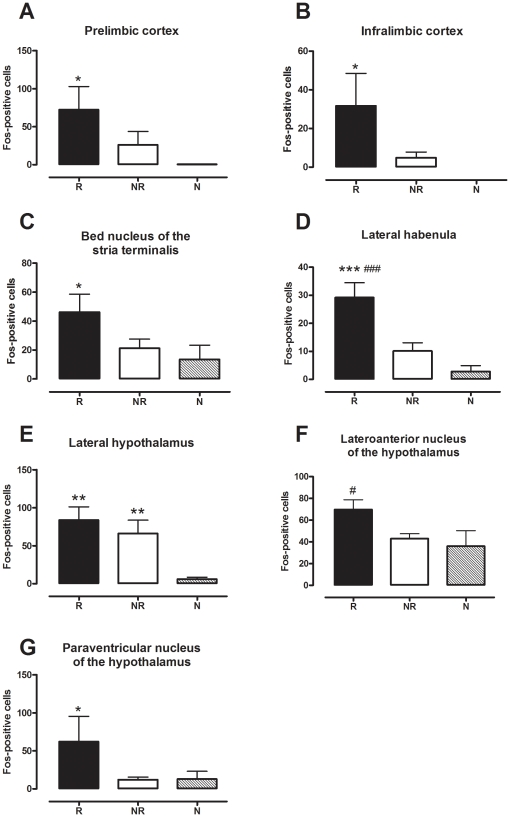
Fos-positive nuclei in reinstating (R) versus non-reinstating (NR) mice and naïve mice (N) (*n* = 5–8 per group). Data are expressed as mean of the number of Fos-positive nuclei for that group (± SEM). (a) Lateral habenula (bregma 1.70); *** *p*<0.001 compared to naïve group, ### *p*<0.001 compared to NR group (Kruskal-Wallis one-way ANOVA on ranks with Dunn's *post hoc* analysis. (b) Bed nucleus of the stria terminalis (bregma 0.14), (c) and (d) cortical regions (bregma 1.70); * *p*<0.05 compared to naïve group (Kruskal-Wallis one-way ANOVA on ranks with Dunn's *post hoc* analysis). (e) Lateral hypothalamus (bregma -1.46); ** *p*<0.01 compared to naïve group (Kruskal-Wallis one-way ANOVA on ranks with Dunn's *post hoc* analysis). (f) Lateroanterior nucleus of the hypothalamus (bregma -0.70-0.92); # *p*<0.05 compared to NR group (one-way ANOVA with SNK post tests). (g) The paraventricular nucleus of the hypothalamus (PVN; bregma -0.70-0.92); * *p*<0.05 compared to naïve group (Kruskal-Wallis one-way ANOVA on ranks with Dunn's *post hoc* analysis).

The question remained as to the nature of the differences between R and NR mice which determined their differing reinstatement propensity. Expression of the protein product of the neuronal activity marker *c-fos* was measured in number of brain regions in order to gain insight into possible neural substrates driving reinstatement propensity. Upon statistical analysis of immunohistochemical data one region was identified in which the activation was significantly higher in R mice as compared to both NR and naïve mice, the lateral habenula (figure 8a,b and 9a). Figure 8 provides representative photomicrographs of Fos immunostaining of this region in R and NR mice. Analysis by Kruskal-Wallis one-way ANOVA on ranks revealed significant differences between the two groups (*p*<0.001). Dunn's post tests revealed that the mean number of Fos-positive nuclei was higher in the lateral habenula in R mice as compared to NR mice and naïve mice (see figure 9a).

Analysis of data for the lateroanterior nucleus of the hypothalamus (anterior part of the anterior hypothalamus; LAH) also revealed significantly higher Fos expression in R mice as compared to NR mice (*p*<0.05) but not naïve mice (*p*>0.05) (figure 9b). This was revealed by one-way ANOVA with SNK *post hoc* analysis. Despite this significant result in the anterior region of this structure, there was no difference between groups in the anterior hypothalamus proper (*p*>0.05, data not shown), demonstrating the regional specificity of this finding.

Analysis by Kruskal-Wallis one-way ANOVA on ranks followed by Dunn's post tests revealed a number of regions where Fos expression was significantly enhanced in R mice but not NR mice as compared to naïve mice. This included cortical regions such as the prelimbic (*p*<0.05) and infralimbic (*p*<0.05) cortices (see figure 9c,d), the bed nucleus of the stria terminalis (BNST, *p*<0.05, dorsal and total, see figure 9b for total), and hypothalamic nuclei such as the paraventricular nucleus of the hypothalamus (PVN, *p*<0.05; figure 8c,d and 9g). As shown in figure 9e, only one region was significantly activated in both R and NR mice as compared to naïve mice, the lateral hypothalamus (*p*<0.01).

### Correlation between Fos expression and reinstatement behaviour

**Figure 10:**
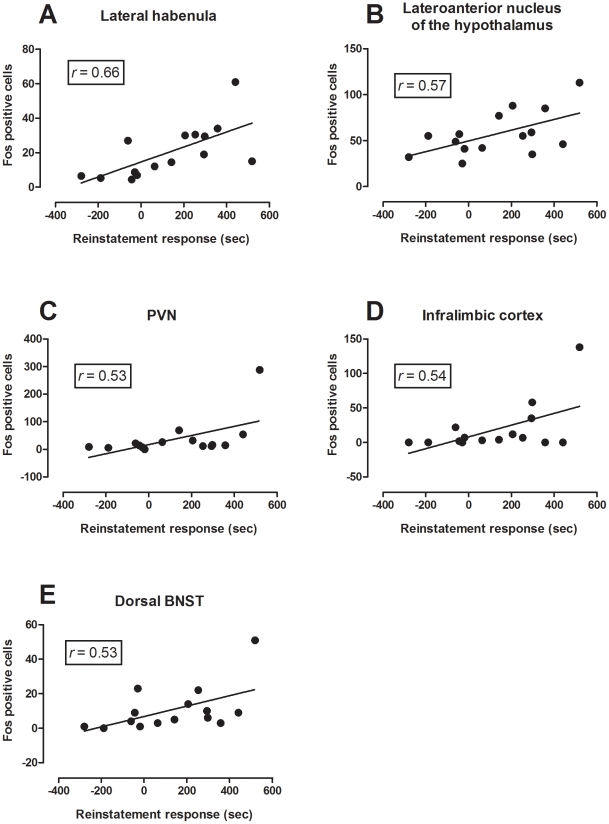
Scatter plots showing the correlation between reinstatement behaviour and Fos expression in the five brain regions where significant correlations were found (*n* = 15). Reinstatement preference score  =  time spent in drug-paired side minus time spent in saline-paired side on test day. Fos-positive cells  =  mean of the number of Fos-immunoreactive cells counted for that region (± SEM). The line represents significant linear correlation (*p*<0.05) between Fos-positive cells and reinstatement score as measured by Pearson's correlation test.

The preferential activation of both the lateral habenula and the LAH in R mice as compared their NR counterparts was supported by correlation data. Both regions demonstrated a positive correlation between Fos expression and reinstatement behaviour. For the lateral habenula (*r* = 0.66) and the LAH (*r* = 0.57) these positive correlations were significant as determined by the Pearson test for correlation (*p*<0.05, figure 10a, b).

Of all the brain regions which were significantly more activated in R mice as compared to naïve mice three regions, the PVN (*r* = 0.54), the infralimbic cortex (*r* = 0.54) and the dorsal BNST (*r* = 0.53) demonstrated a significant correlation between Fos expression and reinstatement behaviour as determined by the Pearson correlation test (*p*<0.05; figure 10c–e). Other regions which were significantly more activated in R mice as compared to naïve mice and displayed a positive correlation with reinstatement data that did not reach significance included the prelimbic cortex (*r* = 0.47) and lateral hypothalamus (*r* = 0.23).

## Discussion

This study is the first to specifically identify possible nuclei which underpin the propensity of mice to exhibit a robust reinstatement of a previously extinguished CPP to cocaine. A main finding of this study is that the lateral habenula is implicated in the circuitry mediating cocaine-primed reinstatement of cocaine-induced CPP. Thus, neural activation following primed reinstatement was increased in the lateral habenula of R mice over both NR and naïve mice. In addition, a significant correlation was found between the propensity to reinstate and activation of this structure. Significant correlations were also found between reinstatement behaviour and neural activation in the infralimbic cortex, BNST and PVN; these structures also showed an increased pattern of activation in R mice as compared to naïve mice, which was not present in NR mice. Another key finding from this study is that the propensity of mice to reinstate was not correlated with the propensity to sensitize to a challenge dose of cocaine or the extent of CPP originally observed. This supports the hypothesis that these aspects of addictive behaviour are likely driven by fundamentally different neural processes.

### Comparisons between reinstatement behaviour and other behaviours

The observation that R and NR mice similarly sensitized to the locomotor activating properties of cocaine despite their differing reinstatement behaviour is an important finding. Behavioural sensitization has been proposed to be analogous to the neural sensitization which occurs with repeated drug use as a result of drug-induced plastic changes in the brain [40], [41]. These neuroadaptations are said to endow an excessive attribution of incentive salience to both the drug and drug-associated stimuli, making addicts compulsively ‘want’ to take drugs again. Hence this process of ‘incentive sensitization’ is proposed to underlie the transition to compulsive drug use and subsequent persistent vulnerability to relapse which occurs in drug addicted individuals [40], [41]. In line with this theory, R mice in this study should therefore have exhibited increased sensitization as compared to NR mice. This was not the case. Thus, contrary to what should be predicted from the incentive-sensitization hypothesis, mice that did not reinstate (NR mice) still sensitized to the psychomotor effects of cocaine. This is in contrast to a previous study which found an association between reinstatement of operant self-administration and sensitization in rats [42]. In this study cocaine trained rats reinstated to an amphetamine prime and exhibited cross-sensitization in response to the same dose of amphetamine. Conversely, cocaine trained rats did not reinstate to a heroin prime or exhibit cross-sensitization to this priming dose of heroin. Comparisons between these data and the current findings are potentially difficult as the proposed association was indirect, involving multiple reinforcers and not supported with correlation analysis. However, the possibility is raised that the relationship between reinstatement and sensitization could differ depending on the paradigm (CPP versus operant). Indeed, reinstatement of conditioned approach in CPP is potentially quite different, both neurobiologically and behaviourally, from reinstatement of operant responding. Despite this, the findings of the current study provide the first direct evidence that reinstatement behaviour is not necessarily associated with sensitization. In addition, the original CPP displayed by R and NR mice subsequent to cocaine conditioning was similar and no correlation was found between the strength of this CPP and the extent of reinstatement after extinction. This demonstrates that not only sensitization, but also the primary rewarding effects of drugs are seemingly dissociable from the propensity to reinstate in this paradigm. Future studies should assess additional behavioural parameters such as impulsivity and anxiety in order to determine the individual differences that contribute to differential reinstatement propensities. Given that no apparent behavioural differences were found between R and NR mice, immunohistochemical analyses helped elucidate region-specific differences in neuronal activation in order to identify the neuroanatomical substrate(s) possibly driving reinstatement behaviour.

### Fos expression: methodological considerations

Fos expression patterns observed in the brains of NR and R mice can potentially result from a combination of some or all of the following stimuli: sensitization to the challenge dose of cocaine, the effects of acute administration of the cocaine prime and/or the cocaine-associated context and subsequent reinstatement behaviour. This means the critical data in this study are not the pattern of neuronal activation in general, as this could be attributed to any one or more of those stimuli, rather the specific differences in expression between the R and NR groups. R and NR mice exhibited identical patterns of locomotor activity; including sensitization to the cocaine prime during the test session. Hence, differences in Fos expression, over and above any background ‘noise’ resulting from drug and/or context, can be correlated with reinstatement behaviour between R versus NR mice.

### Fos expression in brain regions: association with reinstatement

Based on findings from multiple experiments using the extinction-reinstatement model, a ‘final common pathway’ for drug-seeking has been proposed, as that projecting from the medial prefrontal cortex (mPFC) to the nucleus accumbens core [43]. This pathway however, is based on findings obtained via operant studies [44], [45]. Nevertheless, activation of cortical regions in R mice as found in the current CPP study supports this incentive-motivational circuit. The prelimbic cortex specifically has been implicated in primed reinstatement of cocaine-induced CPP [46]. Activation of the prelimbic cortex in R mice is therefore consistent with a role for this component of the mPFC in drug-primed reinstatement behaviour. The role of the infralimbic cortex, on the other hand, is less clear as its involvement in the reinstatement of CPP has not been assessed. Fos expression studies support a role for the infralimbic cortex in cocaine-seeking behaviour as increased Fos expression in the infralimbic cortex is observed as a result of context-induced reinstatement (‘renewal’) of cocaine-seeking [47]. In addition, the infralimbic cortex is activated in response to exposure to cocaine-associated cues and resultant cocaine-seeking behaviour [48]. These data are challenged by studies which implicate the infralimbic cortex in the extinguished response and hence the inhibition of cocaine-seeking [49], [50], [51], [52]. However, Fos expression in the infralimbic cortex was positively correlated with reinstatement behaviour in the current study, indicating that this region is indeed activated and the extent of activation is related to the extent of the reinstatement of the conditioned approach to the CS. Discrepancies between these findings suggest that the role of the infralimbic cortex in reinstatement behaviour is complex, and probably contingent upon the modality of reinstatement (stress, prime, context) and/or the paradigm in question (operant, CPP).

Both nucleus accumbens core and shell were activated in R and NR mice, presumably due to the effects of the cocaine prime (not shown). Another limbic structure, the BNST, was activated in R mice only and a positive correlation was observed between the reinstatement of CPP and Fos expression in this region. The BNST has been shown to be critical in mediating stress-induced, but not cocaine primed, reinstatement of cocaine-seeking in an operant paradigm [53], [54], [55]; whereas inactivation of the BNST prevents both prime and cue-induced reinstatement of heroin-seeking [56]. The present results suggest that the BNST, particularly the dorsal region, may play a role in reinstatement of CPP induced by a cocaine prime. Interestingly, the BNST receives projections from the infralimbic cortex [57], which also displayed a significant correlation between reinstatement behaviour and activation.

The lateral habenula is uniquely positioned both anatomically and functionally to participate in circuits involved in emotion, motivation and cognition [58], [59]. Sites within the habenula support self-stimulation [60], [61] and lesions of the habenula attenuate brain stimulation reward [62]. Recent interest in the lateral habenula stems from an elegant experiment by Matsumoto and colleagues using monkeys which showed that neurons in the lateral habenula are activated by non-reward predicting stimuli and inhibited by reward-predicting stimuli [63]. This led the authors to propose that this region is responsible for the communication of negative reward signals to midbrain dopamine neurons.

Despite the emerging role for the lateral habenula in mediating reward signalling, this structure has been largely ignored in investigations of the neurobiology underlying reinstatement behaviour. The current study provides the first indication of a possible role for the lateral habenula in mediating this behaviour. Interestingly, although previous research indicates that lateral habenula neurons signal negative reward, in this study activation was strongly associated with positive reinstatement behaviour. This suggests a differential role for the lateral habenula in mediating reward versus reinstatement behaviour. In support of current findings, increased Fos expression in the lateral habenula is increased after cue-induced heroin-seeking in an operant paradigm [64]. In addition, two mapping studies reported enhanced Fos expression in the lateral habenula in response to a cocaine-paired environment [35], [65].

Hypothalamic nuclei that displayed significantly enhanced Fos expression in R mice included the PVN, the LAH (lateroanterior nucleus of the hypothalamus) and the lateral hypothalamus. Of these the PVN and LAH showed a significant correlation between activation and reinstatement behaviour. The PVN constitutes the central part of the hypothalamo-pituitary-adrenal (HPA) axis and contains corticotrophin releasing factor (CRF), oxytocin and vasopressin, all of which have been implicated in reinstatement behaviour. Increased expression of Fos has also been found in the PVN following exposure to an environment previously paired with cocaine administration and a recent study showed increases as a result of exposure to ethanol-associated cues and subsequent cue-induced ethanol seeking in an operant paradigm [66].

The LAH has been implicated in both the appetitive and consummatory aspects of male sexual behaviour [67], as well as attack and aggressive behaviour [68], [69]. The LAH was activated significantly more in R mice as compared to NR mice, but not naïve mice. In addition, a significant correlation between this activation and reinstatement behaviour was observed. Interestingly, cocaine has been shown to induce hyperdefensive behaviour in rats [70], [71]. One may speculate that R mice are more responsive to the effects of cocaine, including the effect on this aspect of behaviour. The LAH is specifically innervated by the infralimbic cortex, while the anterior hypothalamus in general is innervated by both prelimbic and infralimbic neurons [57], [72]. Both these components of the mPFC were highly activated in R mice and the pivotal role of the mPFC in driving reinstatement behaviour is well-established [43].

The LAH, PVN and BNST were all activated in R mice and displayed a significant correlation between reinstatement behaviour and activation. The strong activation of these hypothalamic structures as well as the BNST (the ‘stress-reward’ interface) raises the possibility that in R mice a more potent “stress-like” neuroendocrine response occurred. Though further studies are required to assess this possibility, this hypothesis is interesting in the context of the well-established role of stress and HPA axis activation in relapse in humans, as well as in animal models [73], [74].

The lateral hypothalamus is a region of the hypothalamus which has recently gained attention for its role in drug-seeking behaviour [74], [75], [76]. In the current study the lateral hypothalamus was strongly activated in both R and NR mice. It is possible that potential differences between R and NR mice in this case are being masked by activation resulting from cocaine administration. Cocaine priming would result in activation of dopamine D_1_ receptors located in the lateral hypothalamus [77] thus preventing distinction based on drug-seeking.

### Conclusions

The current study demonstrates that the propensity to reinstate a CPP is not associated with either the extent of original CPP or psychomotor sensitization, suggesting a dissociation of these components of addictive behaviour. We also show that reinstatement generally conferred greater Fos expression in cortical and limbic structures previously implicated in drug-seeking behaviour, though a number of regions not typically associated with reinstatement behaviour were also activated. The most significant finding was the activation of the lateral habenula and its correlation with reinstatement behaviour.

## Materials and Methods

### Animals

All experiments were performed in adherence to the Prevention of Cruelty to Animals Act, 1986, under the guidelines of the Australian National Health and Medical Research Council Code of Practice for the Care and Use of Animals for Experimental Purposes in Australia. All experiments were performed with adult male mice on an outbred CD-1 strain housed at the Integrative Neuroscience Facility, Florey Neuroscience Institutes on a 12 h light-dark cycle (light 7am –7pm). Mice were group housed (typically 4 per cage) with nesting material available and free access to food (standard mouse chow) and water.

### Drugs

Cocaine hydrochloride was obtained from Glaxo Australia Pty Ltd (Boronia, Australia) and dissolved in sterile 0.9% saline. Paraformaldehyde was obtained from Sigma Aldrich (St Louis, MO, USA). Depex Mounting Medium was obtained from BDH Laboratory Supplies, Poole, Dorset, UK. The primary antibody for c-Fos (rabbit polyclonal) and was obtained from Santa Cruz Biotechnology Inc. (Santa Cruz, CA, USA). The secondary antibody (biotinylated goat anti-rabbit IgG) and streptavidin horse radish peroxidise were obtained from Vector Laboratories, Burlingame, CA, USA. 3,3′-Diaminobenzidine tetrahydrochloride chromagen (DAB) and ammonium nickel (II) sulphate hexahydrate were obtained from Sigma Aldrich (St Louis, MO, USA) and ammonium chloride from May and Baker Ltd (Dagenham, England). Pentobarbital was obtained from Virbac Australia Pty Ltd (Peakhurst, NSW, Australia).

### Conditioned Place Preference

The CPP protocol was modified from Brown *et al*., (2009) [78]. The CPP apparatus (Lafayette Instruments, Indiana, USA) consisted of two main compartments with differences in visual (wall patterns) and tactile (floor texture) cues, separated by a neutral compartment. The light intensity settings were set at 30 (80 lux) within the conditioning compartments and 90 (380 lux) in the central compartment, with these values referring to the settings on the equipment provided. Before each session mice were habituated to the experimental room for at least 30 min. On day 1 (habituation) mice were placed in the central compartment and allowed free access to the entire apparatus. The time spent in each compartment, as well as general locomotor activity, was recorded via horizontal optic sensor beams and specific software for the apparatus (Motor MonitorTM, Kinder Scientific, CA, USA). Locomotor activity was measured as distance moved (cm).

On days 2–9 (the conditioning phase) mice received alternating injections of cocaine (20 mg/kg/*i.p.*) or vehicle and were immediately confined into one of the two conditioning compartments for 30 min. A combination of unbiased and biased allocation was used. More specifically, mice with a neutral preference (45–55% for either side) were randomly allocated their drug-paired side (unbiased allocation). For the remainder of the mice, the drug was paired with the side which was least preferred (biased allocation).

On day 10 (test day) mice were once again allowed free access to all three compartments. Place preference was determined as a mean positive difference between the time spent in the drug-paired compartment on test day compared to the saline-paired compartment. The compared length of the test session was always 15 min though locomotor activity was measured for longer (30 min) so as to assess the development and expression of sensitization by comparing locomotor activity with that measured during the conditioning sessions.

#### Extinction and subsequent reinstatement of cocaine-induced CPP

CPP was determined as a positive difference greater than 60 s in the time spent in the drug paired compartment on the test day compared to the saline paired compartment. Mice that did not obtain a place preference based on these criteria were removed from the study. Place preference to cocaine was extinguished in a manner previously described [79]. Mice were injected with vehicle and immediately confined to the compartment that was previously paired with cocaine. This occurred for 4 consecutive days and then a test session was conducted (as described above). If the time spent in the drug paired side was within 60 s of the time spent in the saline paired side, mice were deemed extinguished. If mice were not extinguished they were subjected to two additional extinction sessions before another test session. This process was repeated until mice satisfied extinction criteria. All but 2 mice had extinguished after 8 extinction sessions. After 12 extinction sessions (and 7 test sessions) these 2 mice still had a robust place preference for the cocaine-paired side and were therefore excluded from the reinstatement component of the study. Reinstatement was performed the day following extinction. Reinstatement sessions were identical to the test session except mice were injected with a cocaine prime (10 mg/kg, *i.p*; half the conditioning dose) immediately prior to being placed in the central zone of the CPP chamber with free access to all compartments. Locomotor activity was measured for 30 min to enable comparison to the conditioning sessions. This data was normalised to velocity (cm/s^−1^) to account for the differences in the area mice had access to during these sessions.

Mice were deemed reinstated if they spent greater than 60 s in the cocaine-paired compartment compared to the time spent in the saline-paired compartment. Mice were divided into those which did reinstate (R mice) and those which did not reinstate (NR mice) based on this criterion, subsequently the differences between these two groups were assessed via behavioural (*n* = 16, *n* = 11 respectively) and immunohistochemical (*n* = 8, *n* = 7 respectively) analyses. All mice were anaesthetised (pentobarbitone 80 mg/kg *i.p.*) and transcardially perfused 90 min after the reinstatement test session. A control group of 5 naïve mice were acclimatised to the experimental room before being transcardially perfused.

### Tissue preparation

Anaesthetised mice were transcardially perfused with approximately 30 ml phosphate-buffered saline (PBS, 0.1 M; pH 7.4) followed by fixation with approximately 30 ml of 4% paraformaldehyde (PFA) in PBS. The mice were then decapitated and the brain removed and post-fixed overnight in fixative containing 10% (*w/v*) sucrose. Brains were subsequently frozen and sectioned on a cryostat (40 µm) along the coronal plane and sections were floated into 48 well tissue culture microplates containing cryoprotectant solution [80], and stored at −20°C until use.

### Fos immunostaining

Immunohistochemical procedures were performed as previously described [81], [82]. Brain sections from each treatment group were processed simultaneously for each discrete brain region (*n* = 1–3 sections per brain region of interest per mouse from *n* = 5–8 mice per treatment group). Sections were removed from cryoprotectant, washed in 0.1 M PBS (3×10 min), then pre-blocked with 10% normal goat serum (NGS), 0.3% Triton X-100 and 0.1 M PBS for 15 min. Following washing (3×5 min in PBS), sections were incubated with a rabbit polyclonal c-Fos antibody (1∶1000, Santa Cruz Biotechnology, Santa Cruz, CA, USA) in PBS containing 1% NGS and 0.3% Triton X-100 (PBS-NTx) for 48 h at 4°C, with agitation. Sections were then washed and incubated in PBS-NTx containing biotinylated goat anti-rabbit IgG (1∶500, Vector Laboratories, Burlingame, CA, USA) for 1 h, again rinsed, then immersed in PBS-NTx containing streptavidin horse radish peroxidase (1∶500, Vector Laboratories, Burlingame, CA, USA) for 1 h. After washing, sections were reacted with nickel enhanced DAB solution (0.4 M PBS, 0.004% w/v ammonium chloride/ammonium nickel (II) sulphate hexahydrate) for 10 min, and immunoreactivity was then developed by addition of hydrogen peroxide. The reaction was terminated by the addition of 0.1 M PBS to each well. Sections were then transferred into fresh 0.1 M PBS and slide-mounted and coverslipped. Sections which required delineation of subregions such as the nucleus accumbens were lightly counterstained with Neutral Red (0.5% *w/v*, 1 min, Fronine Laboratory Supplies, Taren Point, Australia).

### Histological analysis

Counting of Fos-immunoreactive nuclei was performed unilaterally in each section. Great care was taken to ensure sections were matched at exactly the same anatomical level for each mouse [83]. Brain regions examined included nucleus accumbens core, nucleus accumbens shell (bregma 1.70 and 1.18), prelimbic cortex, infralimbic cortex (bregma 1.70), BNST divided into dorsal and ventral components (bregma 0.14), PVN, LAH (bregma -0.70-0.94), anterior hypothalamus, lateral hypothalamus, and the lateral habenula (bregma −1.46) [83].

Fos-immunoreactive nuclei quantification was conducted either with an Olympus BH-2 microscope or with a stereology L-RGB video capture device analysis system, comprising an upright Leica DMLB-2 microscope, Optronics video unit, running Stereo Investigator 6.00-PR (MicroBrightField Inc., Williston, VT, USA) and using the MicroFire 2.1 B plug-in for Optronics video capture. Quantification was performed in real time with tracing functions of the Stereo Investigator software employed to delineate the particular regions.

#### Statistical Analyses

All data are expressed as mean ± SEM. Statistical analyses were performed using SigmaStat 3.5 and GraphPad Prism 5 software. The effects of group and treatment on CPP or locomotor activity were analysed by repeated measures two-way ANOVA with SNK *post hoc* analyses to compare the different treatment groups and reinstatement states of mice. Time course locomotor data were analysed either by repeated measures two-way or three-way ANOVA with time, group and/or treatment factors, followed by SNK *post hoc* analyses where appropriate. The data for the number of extinction sessions required for mice to reach extinction criteria were not normally distributed so a Mann-Whitney Rank Sum Test was utilised. When comparing totals for locomotor sessions between groups, t-tests were used as there were only two groups for comparison. Correlations between various behavioural data as well as Fos expression were assessed for significance using the Pearson correlation test. Differences were deemed statistically significant if *p*<0.05.

For immunohistochemical data, differences between the groups in terms of mean number of Fos-positive nuclei counted in particular regions was primarily analysed by Kruskal-Wallis one-way ANOVA on ranks as the data were typically not normally distributed. This was followed by Dunn's *post hoc* analyses. On occasions where the data were normally distributed a standard one-way ANOVA was utilised with subsequent SNK *post hoc* analyses. The different treatment groups were naïve mice (N), non-reinstating mice (NR) and reinstating mice (R). Differences were deemed statistically significant if *p*<0.05.
